# Interferometric Synthetic Aperture Microscopy: Computed Imaging for Scanned Coherent Microscopy

**DOI:** 10.3390/s8063903

**Published:** 2008-06-11

**Authors:** Brynmor. J. Davis, Daniel. L. Marks, Tyler. S. Ralston, P. Scott Carney, Stephen. A. Boppart

**Affiliations:** The Beckman Institute for Advanced Science and Technology and The Department of Electrical and Computer Engineering, University of Illinois at Urbana-Champaign, 405 North Mathews Avenue, Urbana, IL 61801, USA; E-mails: bryn@uiuc.edu (B. J. D.); dmarks@uiuc.edu (D. L. M.); tralston@ll.mit.edu (T S. R.); carney@uiuc.edu (P. S. C.)

**Keywords:** Microscopy, Interferometric, Synthetic Aperture, Radar, Optical Coherence Tomography

## Abstract

Three-dimensional image formation in microscopy is greatly enhanced by the use of computed imaging techniques. In particular, Interferometric Synthetic Aperture Microscopy (ISAM) allows the removal of out-of-focus blur in broadband, coherent microscopy. Earlier methods, such as optical coherence tomography (OCT), utilize interferometric ranging, but do not apply computed imaging methods and therefore must scan the focal depth to acquire extended volumetric images. ISAM removes the need to scan the focus by allowing volumetric image reconstruction from data collected at a single focal depth. ISAM signal processing techniques are similar to the Fourier migration methods of seismology and the Fourier reconstruction methods of Synthetic Aperture Radar (SAR). In this article ISAM is described and the close ties between ISAM and SAR are explored. ISAM and a simple strip-map SAR system are placed in a common mathematical framework and compared to OCT and radar respectively. This article is intended to serve as a review of ISAM, and will be especially useful to readers with a background in SAR.

## 1. Introduction

Traditional sensing modalities such as X-ray projection imaging [1], nuclear magnetic resonance (NMR) spectroscopy [2, 3], radar [4] and focused optical imaging [5] rely primarily on physical instrumentation to form an image. That is, the instrument is constructed so that the resulting relation between the object of interest and the collected data is sufficiently simple so as to allow data interpretation with little or no data processing. However, for more than 40 years the performance of microelectronic devices has improved exponentially, as famously quantified, in part, by Moore's law [6]. The resulting abundance of powerful computing resources has been a great boon to almost every area of science and technology, and has transformed sensing and imaging. When significant computational data processing is added to an imaging system, the effect of the physical sensing may be mathematically inverted, allowing the use of instruments with more complicated, multiplex object-to-data relations. The resulting sensing systems provide new imaging modalities, improved image quality and/or increased flexibility in instrument design. This coupling of sensing instrumentation and physically-based inference is often known as computed imaging.

The application of computed imaging techniques to the imaging and non-imaging sensor systems listed above has been revolutionary: X-ray projection imaging has evolved into computed tomography [7, 8]; the contrast mechanisms of NMR spectroscopy form the basis of magnetic resonance imaging [9]; radar has led to synthetic aperture radar (SAR) [10–12]; while the subject of this article, ISAM [13–20], is an example of computed imaging in focused optical systems. Computed imaging techniques also appear in nature—perhaps the most ubiquitous example of what can arguably described as computed imaging is the stereoptic human visual system, where a pair of two-dimensional images (one collected by each eye) are processed in the brain to give depth perception [21]. These examples of computed imaging are far from forming an exhaustive list—the field is large field and growing. Other examples include array-based radio astronomy [22], diffusion tomography [23–25] and positron emission tomography [26]. New applications and contrast mechanisms are still being discovered and the escalation of available computational power is allowing increasingly difficult inverse problems to be solved. For example, the recent explosion of activity in compressive sampling has already brought certain problems in analysis, inference and reconstruction, thought to be intractable, into the realm of tractable problems [27, 28]. Instruments employing compressive sensing not only draw inferences from data using a physical model, they exploit statistical redundancy in the description of the object to significantly decrease the amount of data required, e.g. [29].

This article is focused specifically on ISAM imaging technologies. In addition to the broad commonality ISAM has with other computed imaging techniques, it has strong physical and mathematical connections to a family of instruments including SAR, synthetic aperture sonar [30–32], seismic migration imaging [33, 34] and certain modalities in ultrasound imaging [35, 36]. All of these systems apply computed imaging to multi-dimensional data collected using both spatial diversity and a time-of-flight measure from a spectrally-broad temporal signal. In this article ISAM and SAR are cast in the same mathematical framework, with similarities and differences between the two systems discussed throughout.

In the following section, OCT, the forerunner of ISAM, is described. In Sec. 3 a general framework for ISAM, OCT, SAR and radar is developed. The distinctions between the ISAM/SAR and OCT/radar models are discussed within this framework in Sec. 4. In Sec. 5 it is shown how the models used lead to a simple Fourier-domain resampling scheme to reconstruct the imaged object from the collected data. Simulated and experimental results are shown in Sec. 6, while alternative ISAM instrument geometries are briefly discussed in Sec. 7. Conclusions and references appear at the end of this article.

## 2. Optical Coherence Tomography

An obvious distinction between ISAM and SAR is the spectrum of the electromagnetic field used to probe the sample—ISAM operates in the near infrared (IR), while most SAR systems operate in the radio spectrum. Probing in the near-IR allows the formation of an image with resolution on the order of microns. Additionally, in many biological tissues the near-IR spectral band is primarily scattered rather than absorbed [37], allowing greater depth of penetration than at other wavelengths. Near-IR light backscattered from an object can be used to form a three-dimensional image using OCT [38–41]. Since the image is formed based on the natural scattering properties of the object, OCT and related methods are non-invasive and non-perturbing, c.f., methods such as histology (which requires destruction of the sample) or fluorescence microscopy (which requires staining of the object).

**Figure 1.**
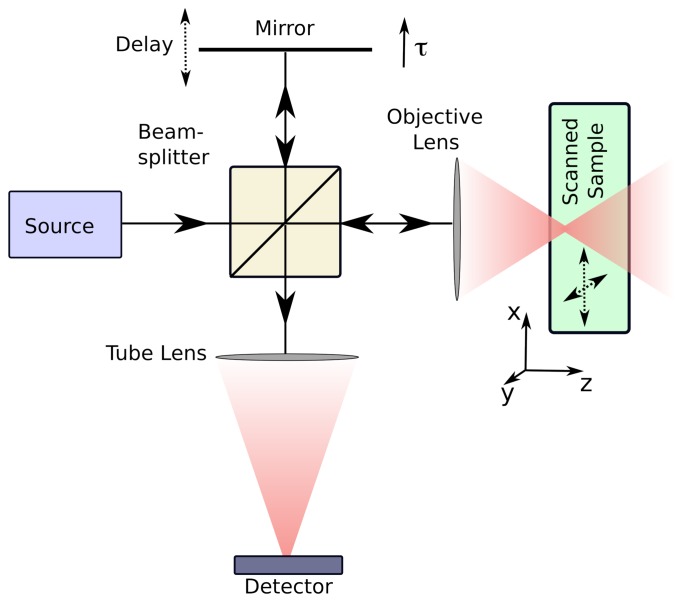
A basic illustration of an OCT system. Light traveling in one arm of a Michelson interferometer is focused into the sample. The length of the reference arm can be adjusted using a moveable mirror. The reference light and the light backscattered from the sample interfere at the detector.

OCT combines interferometry, optical imaging, and ranging. Due to its sensitivity to wavelength-scale distance changes, interferometry has been an important tool in physics (e.g., Young's experiment [42] and the Michelson-Morley experiment [43]) and is now widely applied using many techniques [44]. OCT can be implemented in a Michelson interferometer arrangement as shown in Fig. 1. The focusing optics localize the illumination and collection operations around a transverse focal point. This focal point is scanned in two (transverse) dimensions across the sample. Interferometry with a broadband source is used to image the sample along the third, axial, dimension. The coherently backscattered light and the reference light only interfere for backscattering from a narrow axial (depth) region, of length *L_c_*, determined by the statistical coherence length of the source (see [Ref. 45], Sec. 4.2.1). That is, *L_c_* is inversely proportional to the source bandwidth. The interferometric signal is then obtained as a function of axial position by altering the length of the reference arm for each point in the transverse scan. In this manner optical coherence ranging is used to construct a three-dimensional image.

**Figure 2.**
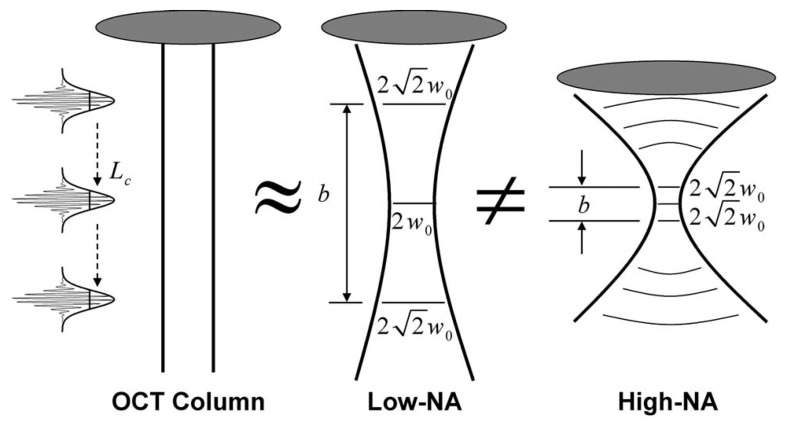
Illustration of focusing in OCT and the trade-off between depth of focus and resolution (figure adapted from [Ref. 17]). In OCT the light is implicitly assumed to be perfectly collimated in a pencil beam. In reality the light must diverge away from the focus. In low numerical aperture systems the beam with *ω*_0_ and the depth of focus *b* are both large. In high numerical aperture systems a tight focal width implies a small depth of field. Axial resolution depends on the coherence length, *L_c_*, of the broadband source.

As described above, depth discrimination in OCT is achieved via coherence gating, while transverse resolution is achieved using focusing optics. Ideal focusing optics would produce a thin collimated beam in the sample, described as a pencil beam in Fig.2.These ideal optics may not be physically realized, as the propagation laws of electromagnetic radiation prohibit beams that are both perfectly collimated and localized. For focusing systems, the beam is often quantified using a scalar Gaussian beam model [46], within which the depth of focus *b* (i.e., the axial depth over which the beam is approximatetly collimated) is proportional to the square of the minimum width *ω*_0_ of the beam. As illustratted in Fig. 2, this relationship between *ω*_0_ and *b* implies that the resolution, which improves with decreasing *ω*_0_, and the depth of focus are competing constraints in OCT. When the coherence gate is set to image planes outside of the depth of focus, the transverse resolution suffers as the beam becomes wider.

ISAM uses computational imaging to overcome the trade-off between depth of focus and resolution. By accurately modeling the scattering processes and the data collection system, including the defocusing ignored in OCT image formation, the scattering properties of the object can be quantitatively estimated from the collected data. As in SAR, diffraction-limited resolution is achieved throughout the final image. for both ISAM and SAR the key to this capability is the coherent collection of a complex data set.

Interferometric microscopes [47], such as OCT systems, give holographic data, i.e., the phase of the backscattered light can be recovered from the raw data. This is a substantial advantage over standard non-interferometric systems where the phase information is lost at detection. This holographic data collection is analogous to the coherent data collection used in SAR systems. Indeed, parallels between SAR and holographic microscopy were recognized and discussed in a series of papers [48-50]. In both ISAM and SAR, the collection of complex coherent data allows the numerical implementation of advantageous operations that would be prohibitively difficult to implement physically. In SAR the multiple along-track range profiles collected from a small aperture can be used to synthesize an aperture corresponding to the whole along-track path. In ISAM, multiple complex OCT range profiles can be computationally reconstructed so that all planes appear simultaneously in-focus, i.e., the blurred out-of-focus regions seen in OCT can be brought into focus numerically.

## 3. General Framework

**Figure 3.**
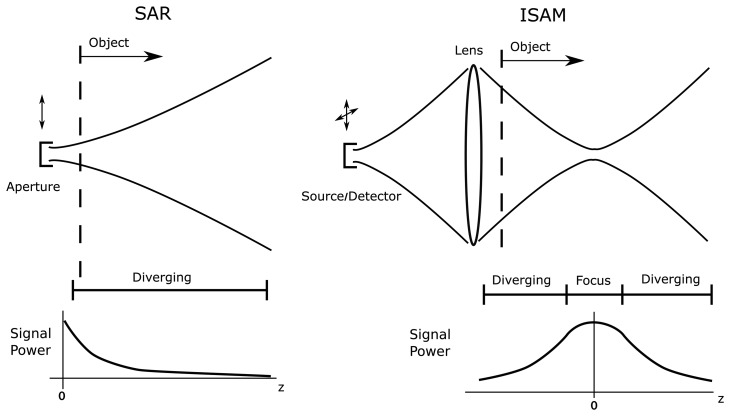
An illustration of the differences between the data acquisition geometries in SAR and ISAM. SAR involves a one-dimensional scan track, while ISAM scans over a plane. Unlike SAR beams, ISAM fields include a region within the object that is in focus. Note that the same aperture is assumed for both transmission and reflection in SAR; similarly the source is imaged onto the detector by the reference arm in ISAM (see Fig. 1). This figure is adapted from [Ref. 51].

In both SAR and ISAM an electromagnetic wave is used to probe the object, the detection apparatus is scanned in space, and a time-of-flight measurement is used to image an additional spatial dimension. Thus, in a fundamental sense, the connection between the data and the object is determined by the same physical laws in either case. This analogy can also be extended to other wave-based techniques such as ultrasound and seismic migration imaging. In this section a general model for radar, SAR, OCT and ISAM techniques is presented. While there are significant differences in system scale and operation, see Fig. 3, the analogy between SAR and ISAM is sufficiently strong to allow a common mathematical description.

As shown in Fig. 3, both SAR and ISAM systems involve a translation of the aperture. This aperture position will be described by a vector ***ρ***, while the vector r describes the position in the imaged object. In the SAR case, a linear-track strip-map system is considered so that the detector is moved along points ***ρ*** = [*x*, 0, 0]*^T^* (superscript *T* indicates a transpose) and the object may be imaged at points in a plane r = [*x*, 0, *z*]*^T^*. In OCT and ISAM the data are collected as a function of two spatial variables in order to image a three-dimensional volume, so that the detector ranges over ***ρ*** = [*x*, *y*, 0]*^T^* and the object may be imaged for r = [*x*, *y*, *z*]*^T^*. Throughout this work a vector will be denoted by bold type, while the corresponding scalar magnitude is given in plain type, e.g., *r* is the magnitude of the vector r. The Fourier transform kernel is exp(*iωt*) for time domain signals and exp(−*i***k** · **r**) for spatial domain signals, so that the complex plane wave exp [*i*(**k**_0_ ·**r** − *ω*_0_*t*)] is a delta function centered on (**k**_0_, *ω*_0_) in the Fourier domain.

### 3.1. The Back-Scattered Field

Consider the scattered field returned to the aperture when the aperture is offset from the origin by ***ρ***, the object consists of a point scatterer at the position r, and an ideal temporal impulse response is used as input to the aperture. This returned scattered field will be denoted by *ĥ*(**r** − ***ρ***, *t*), where the dependence on **r** − ***ρ*** is indicative of the transverse spatial invariance of the system. Under the assumption linearity and temporal invariance, the response to an arbitrary transmitted waveform *Ê_r_*(*t*) is then,

(1) $${\hat{E}}_{s}(\rho ,t)=\int d^{3}r\int dt^{\prime }\;{\hat{E}}_{r}(t^{\prime })\hat{h}(r-\rho ,t-t^{\prime })\eta (r).$$

The linearity of the system is predicated on the assumption that multiple scattering effects are negligible— this is often known as the first Born approximation (see [Ref. 45], Sec. 7.6.2). The system input *Ê_r_*(*t*) is the transmitted radar pulse for SAR systems and the temporal dependence of the optical plane wave incident on the objective lens in ISAM. The object is described by the reflectivity function *η*(**r**) which, in terms of Maxwell's equations, can be identified as the susceptibility (see [Ref. 52], Sec. 2.3). Note that in Eq. (1) the integration over r has been written in three dimensions, while it is a two-dimensional integration for the SAR system.

It is often convenient to represent the temporal convolution seen in Eq. (1) in the Fourier domain so that,

(2) $$E_{s}(\rho ,\omega )=\int d^{3}r\;E_{r}(\omega )h(r-\rho ,\omega )\eta (r).$$

A caret (ˆ) above a function denotes that the function is represented in the space-time domain, while the absence of a caret denotes a function represented in the space-frequency domain. The fact that *η*(**r**) is not a function of *ω* in Eq. (2) is indicative of an implicit assumption made in Eq. (1). The assumption is that the imaged susceptibility is a constant function of the probing signal frequency, i.e., that the object is not dispersive. This assumption is adequate over sufficiently narrow regions of the spectrum or when the object does not have significant absorbing resonant peaks over the imaging band. This is often true to a good approximation in the biological samples imaged using ISAM.

### 3.2. Signal Detection in Radar

The backscattered field incident on the detecting aperture is represented in Eq. (1). Rather than being used directly, this field is typically processed in radar systems, in a technique known as pulse compression. The most common processing used is a matched filter [53], which can be expressed as,

(3) $${\hat{I}}_{R}(\rho ,\tau )={\int \hat{E}}_{s}(\rho ,t){\hat{E}}_{r}^{*}(t-\tau )dt,$$

where *Î_R_* represents the processed radar data. In Eq. (3), the detected field is filtered with a function matched to the broadcast pulse *Ê_r_* (*t*). Note that, following standard practice, a complex analytic representation of the signals has been employed (see [Ref. 45], Sec. 3.1), so that a one-sided Fourier analysis can be used. Implicit in Eq. (3) is a coherent radar detection system sensitive to both the amplitude and phase of the detected oscillating field *Ê_s_*(***ρ***, *t*).

Expressing Eq. (3) in the Fourier domain and using the description of the scattered field given by Eq. (2), the Fourier-domain SAR data may be written

(4) $$\begin{array}{ll} S(\rho ,\omega ) & =E_{s}(\rho ,\omega )E_{r}^{*}(\omega ),\\ & =A(\omega )\int d^{3}r\;h(r-\rho ,\omega )\eta (r), \end{array}$$

where

(5) $$A(\omega )={\left|E_{r}(\omega )\right|}^{2},$$

represents the spectral power distribution of the source.

### 3.3. Signal Detection in Time-Domain OCT and ISAM

While coherent detection of *Ê_s_*(***ρ***, *t*) is possible at the frequencies used in radar, there exist no detectors capable of directly measuring the amplitude and phase of an optical field. However, the phase is indirectly captured through the use of the interferometer. Accurate control of the amplitude and phase of the probing optical signal presents a further complication. These obstacles are surmounted by using a broadband stochastic source and by relying on the coherence-gating effect to measure the time of flight. As shown below, broadband interferometry in OCT and ISAM essentially produces the same effects as coherent detection and pulse compression in radar.

The response times of optical detectors are generally of such a scale that the measured data can be considered a long-time average over optical time scales. Assuming that the fields in the system are statistically stationary and ergodic (see [Ref. 45], Sec. 2.2), these long time averages can be written as

(6) I^T(ρ,τ)=〈|E^r(t−τ)+E^s(ρ,t)|2〉,=Γrr(0)+2Re{Γsr(ρ,τ)}+Γss(ρ,0),

where *τ* is the temporal delay on the reference arm, $\Gamma_{\alpha \beta }(\rho ,\tau )=\langle {\hat{E}}_{\alpha }(\rho ,t){\hat{E}}_{\beta }^{*}(\rho ,t-\tau )\rangle$ and the brackets 〈 〉 represent an ensemble average. That *Î_T_*(***ρ***, *τ*) does not depend on *t* is ensured by the assumption of stationarity.

Because Γ*_rr_*(0) and Γ*_ss_*(***ρ***, 0) do not depend on *τ* in Eq. (6), they may be removed from the data. It can be seen that the data *Î_T_*(***ρ***, *τ*) depend only on the real part of Γ*_sr_*(***ρ***, *τ*), but by taking multiple measurements that include a phase shift in the reference arm (introduced by, for example, a very small translation of the reference arm) it is possible to recover the full complex function Γ*_sr_*(***ρ***, *τ*) [44].

Using the definition of Γ*_sr_*(***ρ***, *τ*) and Eq. (1),

(7) $$\begin{array}{ll} \Gamma_{sr}(\rho ,\tau ) & =\langle \hat{E}(\rho ,\tau ){\hat{E}}_{r}^{*}(t-\tau )\rangle \\ & =\int d^{3}r\int dt^{\prime }\langle {\hat{E}}_{r}(t){\hat{E}}_{r}^{*}(t-\tau )\rangle \hat{h}(r-\rho ,t-t^{\prime })\eta (r),\\ & =\int d^{3}r\int dt^{\prime }\Gamma_{rr}(\tau -t^{\prime })\hat{h}(r-\rho ,t^{\prime })\eta (r). \end{array}$$

As in Eq. (4), these data can be written in the Fourier domain. The Fourier domain data will again be denoted by *S*(***ρ***, *ω*) so that,

(8) $$S(\rho ,\omega )=A(\omega )\int d^{3}r\;h(r-\rho ,\omega )\eta (r),$$

where, in this case, *A*(*ω*) is the power spectral density of the reference beam, which is found, via the Wiener-Khintchine theorem (see [Ref. 45], Sec. 2.4), as the Fourier transform of Γ*_rr_*(*τ*). This power spectral density and the Fourier intensity of Eq. (5) are both real, nonnegative functions which, for the purposes of the data processing examined here, play the same role describing the *ω* bandwidth of the data.

The identical forms of Eq. (4) and Eq. (8) illustrate the commonalities between radar and OCT. Both can be regarded as linear systems collecting data in *N* − 1 spatial and 1 spectral dimension, in order to estimate a spatial-domain object of *N* dimensions. Note that these data collection models have different integral kernels *h*(**r** − ***ρ***, *ω*) and that simplifying assumptions are made to get to the forms of Eq. (4) and Eq. (8). For example, both multiple scattering and nonlinear object responses have been neglected, and it is assumed that a stable phase relation exists between points collected at different scan locations ***ρ***. This last assumption can become problematic in both SAR and ISAM as small unknown variations in the scan path can disturb the assumed relation between data collected at different locations. In both instruments it is usually necessary to introduce some data preprocessing to address this problem. In SAR systems autofocus algorithms [54] are employed, while in current implementations of ISAM, a known structure (e.g., a coverslip boundary) is placed in the object and used as a phase reference point [18, 55]. Such techniques are not necessary in OCT and radar, where phase stability is not required for computed imaging and only the magnitude of the data is typically displayed.

### 3.4. Signal Detection in Fourier-Domain OCT and ISAM

The instrument modality described in the section above and illustrated in Fig. 1 is known as time-domain OCT or time-domain ISAM. It is however, possible to collect the data directly in the frequency domain, in a methodology known as Fourier-domain OCT [56]. In this system the reference mirror seen in Fig. 1 is fixed and the detector replaced with a spectrometer. Fourier-domain OCT eliminates the need for scanning the reference mirror position and has significant advantages in terms of image acquisition time and/or signal-to-noise ratio (SNR) [57, 58]. A complementary principle is applied in Fourier transform infrared spectroscopy [59, 60], where spectral information is measured using interferometric time-domain measurements.

In Fourier-domain OCT or ISAM the collected data are,

(9) $$\begin{array}{ll} I_{F}(\rho ,\omega ) & =\langle {\left|e^{i\omega \tau_{0}}\;E_{r}(\omega )+E_{s}(\rho ,\omega )\right|}^{2}\rangle ,\\ & =\langle {\left|E_{r}(\omega )\right|}^{2}\rangle +2Re\left\{e^{-i\omega \tau_{0}}\langle E_{s}(\rho ,\omega )E_{r}^{*}(\omega )\rangle \right\}+\langle {\left|E_{s}(\rho ,\omega )\right|}^{2}\rangle , \end{array}$$

where *τ*_0_ represents the fixed delay on the reference arm. Note that the Fourier-domain reference and sample fields appearing above are spectral domain representations of random processes. These Fourier domain representations are assumed to exist, at least in the sense of mean-square stochastic convergence of the Fourier integral [61].

The first term of Eq. (9) is the power spectral density *A*(*ω*) used above. This term is constant in ***ρ*** and typically slowly varying with *ω*, and thus can be removed. The last term is known as the autocorrelation artifact and is often small in comparison to the other terms. For this reason it will be assumed negligible here. Note that there are scenarios in which the autocorrelation term may be significant, and in these cases ISAM processing has been shown to mitigate this artifact via a blurring effect [13].

The Fourier spectrum, *S*(***ρ***, *ω*), appearing in Eq. (4), in the deterministic-field context is analogous to the cross-spectral density for the stochastic field,

(10) $$S(\rho ,\omega )=\langle E_{S}(\rho ,\omega )E_{r}^{*}(\omega )\rangle .$$

This suggests that the remaining term in Eq. (9) be written 2**Re**{exp(-*iωτ*_0_)*S*(***ρ***, *ω*)}, with *S*(***ρ***, *ω*) being the desired complex data. While it is possible to determine the complex value of *S*(***ρ***, *ω*) through multiple measurements with different reference phases (as in the time-domain case), a simpler method may be employed if the reference mirror position is set appropriately. Since the sample generally has a well-defined boundary, it is possible to set the reference arm delay *τ*_0_ to be shorter than the least time-of-flight in sample arm plus the coherence length *L_c_*. When this condition is met, the real and imaginary parts of *S*(***ρ***, *ω*) are related via a Hilbert transform. Using simple Fourier transform computations, it is thus possible to recover the imaginary part of *S*(***ρ***, *ω*) from the real observation given in a single measurement [13, 62].

In this section equivalent detection models have been posed for OCT and radar, as represented by Eq. (4) and Eq. (8) respectively. To understand image formation, the integral kernel *h*(**r** − ***ρ***, *ω*) must be examined. This is done in the following section.

## 4. System Modeling

As shown by Eq. (4) and Eq. (8), the relationship between the object *η*(**r**) and the data *S*(***ρ***, *ω*) can be described by the same linear integral equation in both radar/SAR and OCT/ISAM. The modalities differ only in the specific kernels *h*(**r** − ***ρ***, *ω*) which are determined from physics-based models. This section examines the models used for each modality.

### 4.1. Radar and OCT

As described in Sec. 3.1, the time-domain kernel *ĥ*(**r** − ***ρ***, *t*) is the signal returned from a temporal impulse reflected from a scatterer at position r when the beam scan position is ***ρ***. In OCT and strip-map radar, the transmitted and received beams are limited in the transverse directions by focusing, while the range is determined by the signal time of flight. This leads to the kernel,

(11) $$\hat{h}(r-\rho ,t)=u(r_{∥}-\rho )\upsilon (r_{∥}-\rho )\delta [t-t_{d}(z)],$$

where **r**_‖_ is the transverse component of **r**, *u*(**r**_‖_) describes the width of the illuminating beam, υ (**r**_‖_) describes the width of the detection sensitivity, and *t_d_*(*z*) is the time of flight. The kernel described by Eq. (11) is separable in the transverse and axial coordinates and is therefore consistent with the pencil beam approximation illustrated in Fig. 2.

The temporal delay *t_d_*(*z*) is proportional to the twice depth of the scatterer, as a round-trip time of flight is measured. Thus

(12) $$t_{d}(z)=\frac{2z}{c},$$

where *c* is the speed of light. If the same aperture is used in both transmission and detection, reciprocity [63] requires that *u*(**r**_‖_) = υ (**r**_‖_). Appealing to Eq. (11) and Eq. (12), Eq. (7) becomes,

(13) $$\Gamma_{sr}(\rho ,\tau )=\int d^{3}r\Gamma_{rr}\;\left(\tau -\frac{2z}{c}\right)\;u^{2}(r_{∥}-\rho )\eta (r_{∥},z).$$

This expression relates time-domain OCT data to the imaged object. The object is convolved with a PSF with transverse extent governed by *u*^2^(**r**_‖_) and axial extent determined by Γ*_rr_*(*τ*). This relation is similar to one encountered in radar, where the beam width also determines the transverse resolution and the axial resolution is proportional to the length of the compressed broadcast pulse. As illustrated in Fig. 2, Eq. (13) is only valid within the focal region. Beyond this range, blurring and interference artifacts are observed because of beam spread.

It is convenient to take Eq. (13) into the temporal Fourier domain:

(14) $$S(\rho ,\omega )=A(\omega )\int d^{3}ru^{2}(r_{∥}-\rho )e^{i2k\left(\omega \right)z}\eta (r_{∥},z),$$

where *k*(*ω*) is the wavenumber given by the dispersion relation,

(15) $$k(\omega )=\frac{\omega }{c}.$$

This expression is an alternative representation of the time-domain data and directly describes the information bearing term in Fourier-domain OCT. Comparing Eq. (14) to Eq. (8) reveals that the kernel used for the OCT forward model is given by the expression

(16) $$h(\text{r}-\rho ,\omega )=u^{2}({\text{r}}_{∥}-\rho )e^{i2k\left(\omega \right)z}.$$

Reliance on this approximate model limits OCT and radar imaging systems—OCT images are of increasingly poor quality away from the depth of focus, and transverse radar resolution is limited by the beam width and hence the maximum aperture size.

### 4.2. SAR and ISAM

The computed imaging approaches of SAR and ISAM are based on models that more closely approximate solutions of Maxwell's equations. Contrary to the assumptions made in OCT, the transverse and axial system responses cannot be decoupled accurately, due to the beam-spreading illustrated in both Fig. 2 and Fig. 3. The changes in the model are reflected by changes in the kernel *h*(**r** − ***ρ***, *ω*) that appears in Eq. (8). Below, this kernel is analyzed at each temporal harmonic, i.e., the form of the kernel is found at each fixed value of *ω*.

The kernel *h*(**r** − ***ρ***, *ω*) is again separable into the product of illumination and detection patterns as,

(17) $$h(r-\rho ,\omega )=k^{2}(\omega )g(r-\rho ,\omega )\;f(r-\rho ,\omega ).$$

Here the objective lens (ISAM) or transmitting aperture (SAR) produces a field *g*(**r** − ***ρ***, *ω*) in the sample, the detection sensitivity varies with *f* (**r** − ***ρ***, *ω*) and the factor of *k*^2^(*ω*) describes the frequency dependence of scattering. A detailed discussion of this form for *h*(**r** − ***ρ***, *ω*) can be found in [Ref. 14].

When the same aperture is used for both illumination and detection (as is typically the case), reciprocity can again be invoked to show that the illumination and detection patterns are equal. Furthermore, the illumination field *g*(**r** − ***ρ***, *ω*) must obey propagation laws. This means that in a homogenous background medium, the illuminating field can be repressented by a spectrum of plane waves (see [Ref. 52], Sec. 11.4.2),

(18) $$g(r,\omega )=\int d^{2}q_{∥}\;G(q_{∥},\omega )\;\exp\;\{i[q_{∥}\cdot r_{∥}+k_{z}(q_{∥},\omega )z]\},$$

where,

(19) $$k_{z}(q_{∥},\omega )=\sqrt{k^{2}(\omega )-q_{∥}^{2}}.$$

In free space *k*(*ω*) is given by Eq. (15), however more complicated dispersion relations can also be used for dispersive materials [18, 64, 65], where the speed of light depends on *ω*.

The angular spectrum of Eq. (18) must be modified for the two-dimensional SAR system. In SAR a two-dimensional (*x*, *z*) object is imaged, meaning that **r**_‖_ and **q**_‖_ are each one-dimensional. However, the electromagnetic fields present in the system spread in three dimensions. In the simple strip-map SAR system considered here, the SAR aperture track and the object are both assumed to lie in the *x*–*z* plane, i.e., the aperture altitude is neglected. In this geometry the spreading in *y* can be modeled as a [*k*(*ω*)*z*]^−1/2^ decay so that, for the SAR system, Eq. (18) becomes,

(20) $$g_{s}(\text{r},\omega )=\frac{1}{\sqrt{k(\omega )z}}\int dq_{x}\;G_{s}(q_{x},\omega )\;\exp\;\{i[q_{x}x+k_{z}(q_{x},\omega )z]\}.$$

As will be seen subsequently, this difference in dimensionality between SAR and ISAM does not change the nature of the data processing required, only the dimensionality of the processing.

The angular spectra *G*(**q**_‖_, *ω*) and *G_s_*(*q_x_*, *ω*) seen in Eq. (18) and Eq. (20) can be related to the aperture shapes used in ISAM and SAR. In ISAM the focal plane is defined to be at *z* = 0, resulting in the function *G*(**q**_‖_, *ω*) being directly related to the lens aperture. For high numerical aperture lenses the function *G*(**q**_‖_, *ω*) is broad and the beam width at the focus narrow. Aberrations on the lens can be included in the phase of the angular spectrum. A simple model for *g*(**r**, *ω*) is a Gaussian beam [46], where *G*(**q**_‖_, *ω*) is a Gaussian function. More thorough models, e.g., [66], can also be used within the developed framework. In SAR, the *z* = 0 plane is chosen to coincide with the track of the radar aperture. In this case the spectrum *G_s_*(*q_x_*, *ω*) corresponds to the Fourier transform of the aperture profile. A small aperture gives a highly divergent beam.

The forward model used in SAR and ISAM (Eq. (17)) is more accurate than that assumed in radar and OCT (Eq. (16)). In the simple OCT model each point in the data set is associated with a point in the object, as described in Eq. (13). To correct for the out-of-focus blurring described by the more accurate kernel of Eq. (17), mathematical processing must be applied. The appropriate computed imaging algorithm is described in the next section.

## 5. The Inverse Problem for SAR and ISAM

The linear integral equation of Eq. (8) and the expression for the kernel, given in Eq. (17), form the forward model used in SAR and ISAM. This relation describes the dependence of the data on the object. Estimating the object from the data, using the forward model, requires solving the inverse problem. In general this problem may be ill-posed, but with the use of regularization techniques [67-69], an estimate of the object may be found. The quality of this estimate will depend on how much information is passed by the instrument.

Since the ISAM forward model is well defined, the inverse problem can, in principle, be solved using numerical techniques. However, an approximation to the forward model allows a more elegant, and significantly more efficient [20], solution to the inverse problem. This solution is explained in this section.

### 5.1. Transverse Spatial Fourier Representation of the Model

The angular spectrum representations seen in Eq. (18) and Eq. (20) give the transverse spatial Fourier transform of the illuminating field *g*(**r**, *ω*). The model kernel can then be taken to the transverse spatial Fourier domain, denoted by a tilde, by noting that the product seen in Eq. (17) becomes a convolution,

(21) $$\tilde{h}(q_{∥},z,\omega )=k^{2}(\omega )\int d^{2}q_{∥}^{\prime }\;G(q_{∥}^{\prime },\omega )G(q_{∥}-q_{∥}^{\prime },\omega )\exp\;\{i[k_{z}(q_{∥}^{\prime },\omega )+k_{z}(q_{∥}-q_{∥}^{\prime },\omega )]z\}.$$

Comparing Eq. (18) and Eq. (20), it can be seen that the SAR result is similar to the expression above but in one fewer dimension and with a prefactor of [*k*(*ω*)*z*]^−1^.

As a first step towards the solution of the inverse problem, it is useful to recognize that the transverse part of the integral appearing in Eq. (8) is in the form of a two-dimensional convolution. Thus, by taking the two-dimensional (transverse) spatial Fourier transform of the data, the inverse problem may be reduced from a problem involving a three-dimensional integral equation to one of a series of one-dimensional integral equations, i.e.,

(22) $$\tilde{S}(q_{∥},\omega )=A(\omega )\int dz\;\tilde{h}(-q_{∥},z,\omega )\tilde{\eta }(q_{∥},z).$$

### 5.2. Model Approximation in Diverging Regions

As illustrated in Fig. 3, the fields used in SAR and ISAM are divergent away from the *z* = 0 plane. In Eq. (21), this implies that the complex exponential factor in the integrand is rapidly oscillating. Such oscillatory integrals can be approximated using the method of stationary phase (see [Ref. 45], Sec. 3.3). The stationary point occurs when the argument of the exponential has zero gradient, which in this case is at the point ${\text{q}}_{∥}^{\prime }={\text{q}}_{∥}/2$.

Applying the method of stationary phase in two dimensions gives the ISAM result,

(23) $$\tilde{h}(q_{∥},z,\omega )\approx \frac{H_{D}(q_{∥},\omega )}{k(\omega )z}\exp\left\{i2k_{z}\left(\frac{q_{∥}}{2},\omega \right)z\right\},$$

where *H_D_*(**q**_‖_, *ω*) describes the bandwidth of the data (see [Ref. 14] for an exact description of this function). The factor of [*k*(*ω*)*z*]^−1^ appearing above describes the signal decay away from focus. In SAR, the method of stationary phase in one dimension is applied to a kernel based on the angular spectrum of Eq. (20). The result is of the same form as Eq. (23) but with a decay of [*k*(*ω*)*z*]^−3/2^.

### 5.3. Model Approximation in Focused Regions

As seen in Fig. 3, the object in ISAM, unlike in SAR, contains the focused *z* = 0 plane. Around this region the exponential seen in the integrand of Eq. (21) is not highly oscillatory, meaning the method of stationary phase can not be accurately applied. However, it is still possible to approximate the function *h̃*(**q**_‖_, *z*, *ω*) to obtain an elegant inversion [19].

In the focal region, the integrand of Eq. (21) is dominated by the product $G({\text{q}}_{∥}^{\prime },\omega )G({\text{q}}_{∥}-{\text{q}}_{∥}^{\prime },\omega )$. For symmetric apertures, this product will be peaked around the point ${\text{q}}_{∥}^{\prime }={\text{q}}_{∥}/2$. The exponential factor may be expanded in a Taylor series about this point and, since it is slowly varying for small *k*(*ω*)*z*, all but the leading term discarded. The consequent analysis, given in detail in [Ref. 14], then results in an approximation of the form,

(24) $$\tilde{h}(q_{∥},z,\omega )\approx H_{F}(q_{∥},\omega )\;\exp\;\left\{i2k_{z}\left(\frac{q_{∥}}{2},\omega \right)z\right\}.$$

The exponential factor above is the same as for the diverging region.

### 5.4. Reduction to Resampling

The approximated models described above can be substituted into the data model of Eq. (22) to give,

(25) $$\tilde{S}(q_{∥},\omega )\approx A(\omega )H(-q_{∥},\omega )\int dz\frac{\tilde{\eta }(q_{∥},z)}{R(z)}\;\exp\;\left\{i2k_{z}\left(\frac{q_{∥}}{2},\omega \right)\;z\right\},$$

where *H* (**q**_‖_, *ω*) = *H_F_*(**q**_‖_, *ω*) and *R*(*z*) = 1 when considering *z* in the focused region, and *H* (**q**_‖_, *ω*) = *H_D_*(**q**_‖_, *ω*) and *R*(*z*) = *k*(*ω*)*z* (or *R*(*z*) = [*k*(*ω*)*z*]^3/2^ in the SAR case) for *z* in the diverging region. The transition point between these two regimes is discussed in [Ref. 14].

In Eq. (25), *A*(*ω*) *H*(−**q**_‖_, *ω*) act as linear filters on the data. The effects of these filters can be compensated by standard means, such as the Wiener filter [70]. For systems without aberrations, the function *H*(**q**_‖_, *ω*) is slowly varying, as is *A*(*ω*), meaning that it may be acceptable to neglect the effects of *A*(*ω*)*H*(−*q*, *ω*) in many situations.

In either case, the remaining integral in Eq. (25) can be seen to be of the form of a Fourier transform. Consequently,

(26) $$\tilde{S}(q_{∥},\omega )\propto {\overset{\approx }{\eta }}^{\prime }[q_{∥},q_{z}(q_{∥},\omega )],$$

where *η͌′* is the three-dimensional Fourier transform of *η*(**r**)/*R*(*z*), the object with an attenuation away from focus, and

(27) $$\begin{array}{ll} q_{z}(q_{∥},\omega ) & =-2k_{z}(q_{∥}/2,\omega ),\\ & =-\sqrt{4k^{2}(\omega )-q_{∥}^{2}}\cdot \end{array}$$

**Figure 4.**
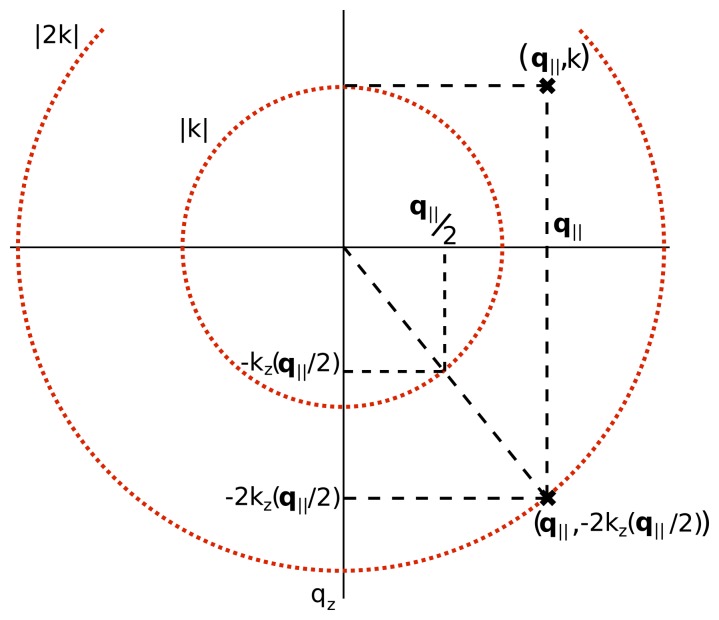
A geometric illustration of the Stolt mapping relating a point [**q**_‖_, *k*(*ω*)] in the Fourier-domain data to a point [*q*_‖_, −2*k_z_* (**q**_‖_/2, *ω*)] in the Fourier-domain object. Note that the *ω* dependence of the displayed quantities has been dropped for convenience. This figure is adapted from [Ref. 14].

This equation describes a Fourier domain warping relating the data and the object. This warping is known as the Stolt mapping and is illustrated in Fig. 4. The Stolt mapping was originally developed in the field of geophysical imaging [71, 72] and is used in Fourier migration techniques. In ultrasonic imaging, Eq. (27) forms the basis of the Synthetic Aperture Focusing Technique (SAFT) [73-76]. The Stolt mapping was also recognized as applicable in SAR [77], where it is typically known as the *ω*−*k* algorithm or the wavenumber algorithm. This work shows the utility of the Stolt mapping in the field of interferometric broadband microscopy.

The equivalent Fourier mapping for OCT, found from the kernel of Eq. (16) and valid only within the focal region, is

(28) $$q_{z}(q_{∥},\omega )=-2k(\omega ).$$

This OCT model describes only a rescaling of the axial coordinate, while the Stolt mapping of Eq. (26) describes the physical effects of out-of-focus beam spreading.

The relation given in Eq. (26) gives a clear indication of how to estimate the object from the collected data *S*(***ρ***, *ω*). This procedure can be summarized as

1. Starting with the complex data *S*(***ρ***_‖_, *ω*), collected as described in Sec. 3, take the transverse spatial Fourier transform to get *S̃*(**q**_‖_, *ω*).
2. Implement a linear filtering, i.e., a Fourier-domain multiplication of a transfer function with *S̃* (**q**_‖_, *ω*), to compensate for the bandpass shape given by *A*(*ω*) *H*(−**q**_‖_, *ω*) in Eq. (25). This step may often be omitted without significant detriment to the resulting image.
3. Warp the coordinate space of *S̃* (**q**_‖_, *ω*) so as to account for the Stolt mapping illustrated in Fig. 4. Resample the result back to a regular grid to facilitate numerical processing.
4. Take the inverse three-dimensional Fourier transform to get an estimate of *η*(**r**)/*R*(*z*), the object with an attenuation away from focus.
5. If required, multiply the resulting estimate by *R*(*z*) to compensate for decay of the signal away from focus.

The operations described above are computationally inexpensive and allow a fast implementation of ISAM processing [20].

## 6. Results

In this section ISAM images are compared to those obtained using standard OCT methods. The high quality of the results obtained validates the calculations made above, while also showing that the approximations made to the forward model in Sec. 5, do not introduce significant error in the solution to the inverse problem.

### 6.1. Simulations

**Figure 5.**
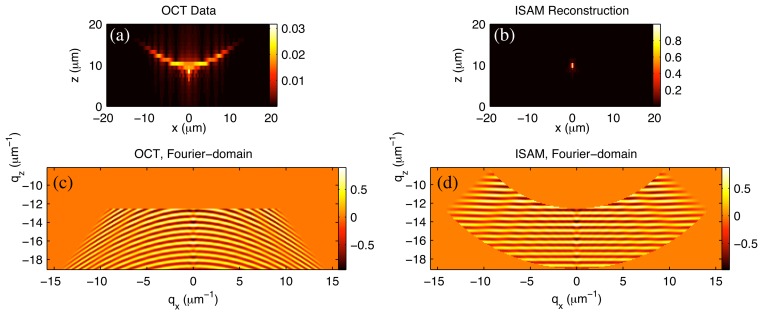
Simulated OCT image from a point scatterer located at (0, 0, 10)*μ*m (a) and the real part of the corresponding Fourier representation (c). The ISAM Fourier resampling takes the data shown in (c) to the reconstruction of (d). The corresponding spatial-domain ISAM reconstruction is shown in (b). The ISAM reconstruction describes the point scatterer accurately, while defocus is clearly observed in the OCT image. Note that the two-dimensional images shown represent one plane of three-dimensional functions. This figure is adapted from [Ref. 51].

Numerical simulations of the ISAM system are useful for providing a theoretical corroboration of the proposed methods in a tightly controlled and well understood environment. In Fig. 5, simulation results are shown for the imaging of an isotropic point scatterer located out of focus on the *z* axis.

The data were produced using the focused vector beam formulation given in [66]. The electromagnetic field defined in that paper is an exact solution to Maxwell's equations, and obeys geometrical-optics boundary conditions on the lens aperture. An objective lens with 0.75 numerical aperture was simulated and light between the wavelengths of 660nm and 1000nm was collected. Further details of this type of data simulation can be found in [Ref. 14].

The magnitude of the spatial-domain OCT data gives a broadly spread and low-amplitude response. Ideally the image would be point-like, corresponding to the point scatterer. The blurring observed is due to the scatterer being in the out-of-focus region. When the OCT image is examined in the Fourier domain, curved phase fronts can be seen. For the offset point scatterer imaged, the Fourier spectrum should have flat phase fronts parallel to the *q_x_*–*q_y_* plane.

The Fourier resampling of ISAM can be seen to take the curved OCT phase fronts to the expected straight lines. When the ISAM image is represented in the spatial domain, the desired high-amplitude, point-like image is seen. These simulations lend strong support to ISAM, as the detailed, vectorial forward model is inverted accurately by a simple Fourier-domain resampling only.

### 6.2. Imaging a Phantom

Beyond simulations, the next step in ISAM validation is to image an engineered object (i.e. a phantom) with known structure. Here the phantom was constructed by embedding titanium dioxide scatterers, with a mean diameter of 1*μ*m, in silicone. This phantom was imaged with a spectral-domain ISAM system employing an objective lens with a numerical aperture of 0.05. A femtosecond laser (Kapteyn-Murnane Laboratories, Boulder, Colorado) was used as a source, to give a central wavelength of 800nm and a bandwidth of 100nm. The resulting focused pattern *g*(**r**, *ω*) can be approximated as a Gaussian beam with a spot size of 5.6*μ*m and a depth of focus of approximately 240*μ*m. Further details of the ISAM instrument and the phantom can be found in [Ref. 18].

**Figure 6.**
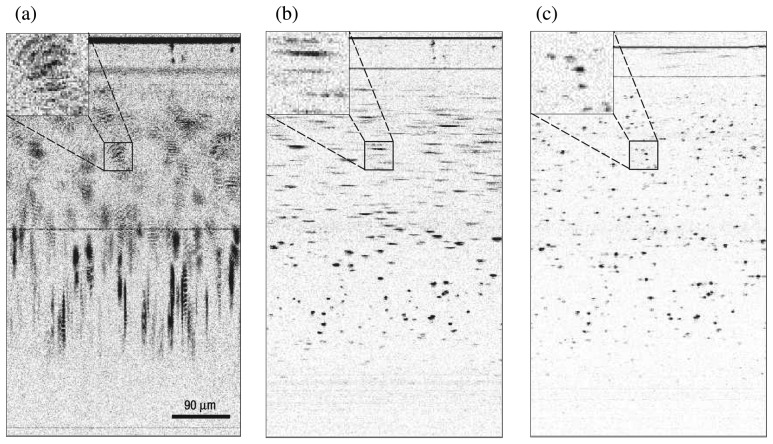
Images of titanium dioxide scatterers—OCT image before dispersion compensation (a), OCT image after dispersion compensation (b), and ISAM reconstruction (c). This figure is adapted from [Ref. 18].

**Figure 7.**
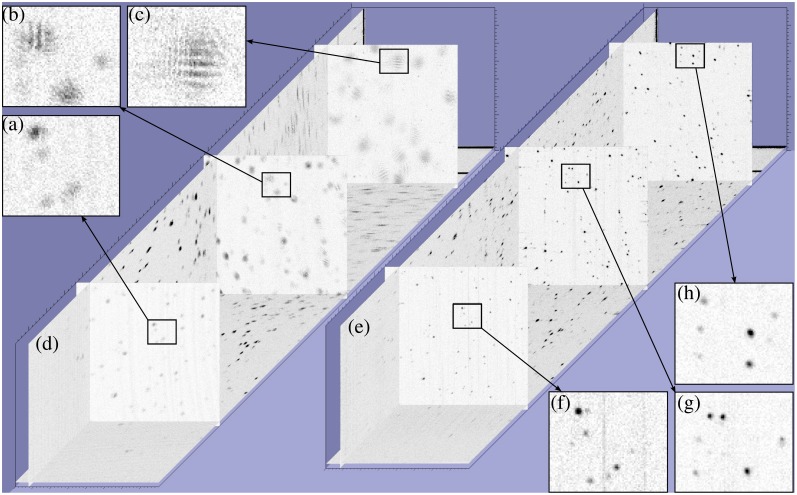
Planar *x*–*y* slices of the OCT image volume (d) and the ISAM reconstruction volume (e). Three planes are shown, with details of extent 80*μ*m×80*μ*m for each. The planes are located at *z* = − 1100*μ*m (c,h), *z* = − 475*μ*m (b,g) and *z* = 240*μ*m (a,f), where *z* = 0 is the focal plane. This figure is adapted from [Ref. 18]

**Figure 8.**
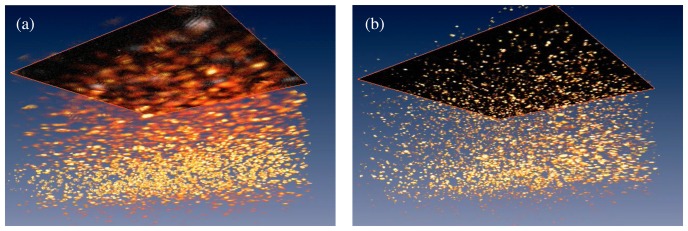
Three-dimensional renderings of the OCT (a) and ISAM (b) images of titanium dioxide scatterers. Out of focus blur can be seen in the OCT image, while the ISAM reconstruction has isotropic resolution. Note that the axial axis has been scaled by a factor of 0.25 for display purposes. This figure is adapted from [Ref. 18].

ISAM processing, including dispersion compensation [64], was applied to the collected data to produce an image. Specific details of the computational implementation can be found in [Ref. 18], while [Ref. 20] gives a thorough general description of ISAM algorithms and computational demands. The raw data and the ISAM reconstruction are shown in Fig. 6 and Fig. 7 (transverse-axial and transverse-transverse planes respectively), with corresponding renderings in Fig. 8.

Out of focus blurring is clearly visible in the collected data. This blurring limits the depth of field in OCT. The ISAM reconstruction can be seen to bring the out-of-focus regions back into focus, as evidenced by the point-like features in the image, which correspond to individual titanium dioxide scatterers. It should be noted that the point-like reconstructions observed are produced by the physics-based computational imaging, not by the use of any assumed prior knowledge of the sample, e.g., [78]. The *x*–*y* details of Fig. 7 provide further insight into the action of the ISAM resampling algorithm. In Fig.7(b) and Fig.7(c) interference fringes can be clearly seen. These result from the simultaneous illumination of two (or more) point scatterers and the consequent interference of the light scattered from each. The reconstructions of Fig.7(g) and Fig.7(h) show that these interference fringes are correctly interpreted as multiple point scatterers in the ISAM reconstruction.

**Figure 9.**
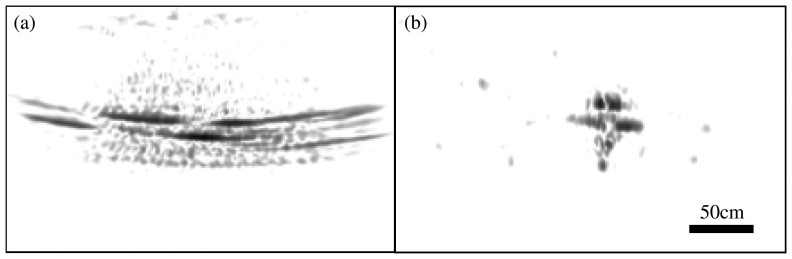
Raw strip-map radar image of a 1:32 scale model of a F14 fighter aircraft before Stolt Fourier resampling (a), and after Stolt Fourier resampling (b).

To further illustrate the SAR-ISAM analogy, ISAM and SAR images are compared below. Strip-map radar and SAR images from a linear rail SAR imaging system [79,80] are shown in Fig.9. This imaging system consists of a small radar sensor mounted on linear rail that is 225cm in length. The radar sensor is moved down the rail at 2.5cm increments, acquiring a range profile of the target scene at each location along the rail. The radar sensor is a linear FM radar system with 5GHz of chirp bandwidth spanning approximately 7.5GHz to 12.5GHz. The chirp time is 10ms, the transmit power is approximately 10dBm, the receiver dynamic range is better than 120dB, and the digitizer dynamic range is 96dB. Range profile data from each increment across the rail are fed into a range-migration SAR algorithm [12], a stolt Fourier resampling, to yield a high-resolution SAR image of the target scene. Raw radar range profile data are similar to out of focus data in coherence microscopy, as seen in Fig. 9(a) for radar and Fig. 6(b) for OCT. The SAR image, after Stolt Fourier resampling, is shown in Fig. 9(b), which is analogous to the ISAM image of Fig. 6(c).

### 6.3. Imaging Tissue

**Figure 10.**
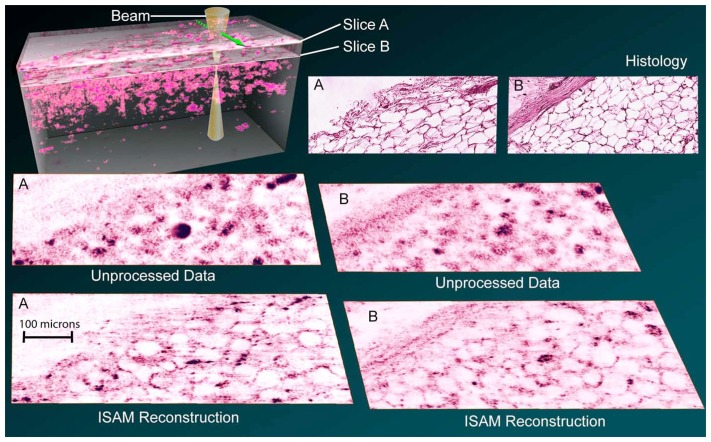
Breast tissue is imaged according to the geometry illustrated in the rendering in the upper left. Data are shown in the *x*–*y* plane for two different values of *z*. Plane A is at *z* = − 643 *μ*m, while plane B is at *z* = − 591 *μ*m. ISAM image reconstruction can be seen to produce a significant improvement in image quality over the unprocessed OCT data in both planes. The ISAM reconstructions exhibit comparable features to histological sections. This figure is adapted from [Ref. 18].

OCT and ISAM are primarily biological imaging methods. As such, the most important capability of ISAM is the imaging of tissue. As described in [Ref. 18], human breast tissue was acquired and imaged with the same ISAM system used to image the titanium dioxide scatterers. Examples of the resulting images can be seen in Fig. 10. Once again, it can be seen that ISAM successfully removes blur and resolves interference artifacts in otherwise out of focus regions.

The improvement observed in the ISAM reconstructions has significant consequences in terms of the diagnostic utility of the images. In the out-of-focus OCT images, the cellular structure is almost entirely lost, while in the ISAM reconstructions, significant features can be seen on the micrometer scale. For example, cell membranes can be recognized, and the boundary between the adipose and fibrous tissue can be clearly seen. There is also a strong correspondence to the histological sections, although embedding, sectioning and staining of the tissue disrupt the sample to some extent. ISAM, unlike OCT, can be seen to allow diffraction-limited imaging at all planes within the sample, rather than just at the physical focus. As a result, significantly more information regarding the tissue can be extracted without increasing the measurement duration or scanning the focal plane. In contrast to the histological images, the structure visible in the ISAM images is observed without destruction of the sample. This suggests ISAM may be particularly useful in applications where in vivo imaging over a large tissue volume is preferable to biopsy.

## 7. Alternate ISAM Modalities

ISAM is a microscopic imaging technique and is implemented on a bench-top scale. This provides significant flexibility in the design of alternative ISAM modalities. In this section some alternative ISAM instruments are briefly discussed.

### 7.1. Vector ISAM

To achieve a maximum-resolution image it is necessary to use the highest possible numerical aperture objective lens (high-numerical-aperture OCT is often known as optical coherence microscopy [81]). For such high-angle lenses the electromagnetic fields present in the system cannot be accurately approximated as scalar fields. Furthermore, it has been shown that the vectorial nature of the high-aperture focused field can be explicitly exploited to probe anisotropic properties of the object, e.g., [82-87]. ISAM can be generalized to vectorial fields [14].

In the vectorial system, scattering from the object is recognized as being dependent on the polarization state of the relevant fields. As a result, the object is not a scalar function *η* (**r**), but a rank-two tensor function of position ***η̄*** (**r**). The illumination and detection patterns, **g**(**r**, *ω*) and **f** (**r**, *ω*), are vectorial, which results in six independent ISAM kernels—one for each possible pair of field directions in illumination and detection. That is,

(29) $$h(r-\rho ,\omega ,\alpha ,\beta )=k^{2}(\omega )g(r-\rho ,\omega ,\alpha )f(r-\rho ,\omega ,\beta ),$$

where *g*(**r** − *ρ*, *ω*, *α*) is an element of the field **g**(**r**,*ω*), and *α* takes on values of *x*, *y* and *z*. The scalar kernel of Eq. (17) is a special case of this expression.

It can be shown that the data then depend on the scattering tensor as [14],

(30) $$S(\rho ,\omega )=\sum_{\alpha \in \{x,y,z\}}\sum_{\beta \in \{x,y,z\}}\int d^{3}r\;h(r-\rho ,\omega ,\alpha ,\beta )\bar{\eta }(r,\alpha ,\beta ),$$

where *η̄*(**r**, *α*, *β*) is an element of the tensor *η̄*(**r**). The scalar case of Eq. (8) is a special case of this expression.

It can be seen from Eq. (29) that *h*(**r** − ***ρ***, *ω*, *α*, *β*) = *h*(**r** − *ρ*, *β*, *ω*, *α*). Symmetry arguments [88], can be used to show the equivalent property, *η̄*(**r**, *α*, *β*) = *η̄*(**r**, *β*, *α*), for the scattering tensor. The effect of each independent element of the scattering potential on the data is therefore described by a distinct kernel.

### 7.2. Full-Field ISAM

Full-field OCT systems [89-93] involve capturing *x*–*y* images sequentially, one frequency *ω*, or delay *τ*, at a time. A similar system has been analyzed for ISAM [15]. In this system the object is illuminated with a *z*-propagating plane wave, so that the illumination pattern is

(31) g(r,ω)=exp[ik(ω)z].

The angular spectrum of the illuminating light is then,

(32) $$G(q_{∥},\omega )=\delta (q_{∥}).$$

The scattered light is collected by an objective lens, so that the detection pattern *f*(**r**, *ω*) is of the same form as *g*(**r**, *ω*) in Eq. (18).

The spatial-domain kernel of Eq. (17), can then be taken into the Fourier domain using the same process as that used to find Eq. (21). That is,

(33) $$\begin{array}{ll} \tilde{h}({\text{q}}_{∥},z,\omega ) & =k^{2}(\omega )\int d^{2}q_{∥}^{\prime }\delta (q_{∥}^{\prime })\;F(q_{∥}-q_{∥}^{\prime },\omega )\;\exp\;\{i[k_{z}(q_{∥}^{\prime },\omega )+k_{z}(q_{∥}-q_{∥}^{\prime },\omega )]z\}\\ & =k^{2}(\omega )\;F(q_{∥},\omega )\;\exp\;\{i[k(\omega )+k_{z}(q_{∥},\omega )]z\}. \end{array}$$

This exact kernel is of the same form as the approximated forward models of Eq. (23) and Eq. (24). As a result, the relationship between the Fourier-domain data and the Fourier-domain object for the full-field ISAM system is of the same form as Eq. (26), but with the new mapping,

(34) $$q_{z}(q_{∥},\omega )=-k(\omega )-k_{z}(q_{∥},\omega ).$$

Thus the inverse problem in full-field ISAM is also solved by a Fourier-domain resampling, albeit on a different grid.

Confocal ISAM is analogous to SAR, and both techniques share the Stolt mapping. The full-field ISAM mapping of Eq. (34) also appears in diffraction tomography [94–96], a technique applied in ultrasound, optical and microwave imaging.

### 7.3. Rotationally-Scanned ISAM

**Figure 11.**
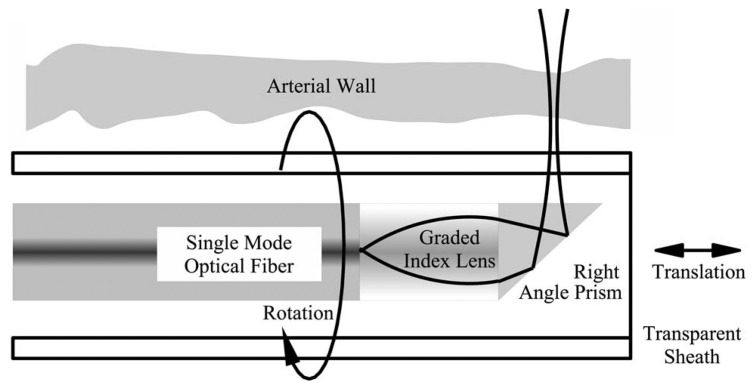
An illustration of the rotationally-scanned ISAM system. A single-mode fiber delivers light to focusing optics which project the beam into the object. The beam is scanned linearly inside a catheter sheath and is rotated about the long catheter axis. This figure is adapted from [Ref. 16].

Rotationally-scanned ISAM [16] is a sensing system compatible with catheter-based imaging, as illustrated in Fig. 11. Rather than scanning the aperture in two coplanar dimensions, the aperture is scanned in one linear dimension (along the catheter) and one rotational dimension (along the azimuthal angle).

The complex analytic signal, *S*(*p*, *θ*, *ω*), is sampled at points given by the displacement p along the y axis and the azimuthal coordinate *θ*, as well the usual frequency *ω*. Taking the Fourier transform with respect to both *p* (argument *ξ*) and *θ* (argument *η_θ_*) results in the data *S̃*(*ξ*, *η_θ_*, *ω*). After some calculation an approximate expression is obtained for the transformed data:

(35) $$\tilde{S}(\xi ,n_{\theta },\omega )=K(\xi ,n_{\theta },\omega )\overset{\approx }{\kappa }(\xi ,n_{\theta },\omega ),$$

where *K*(*ξ*, *η_θ_*, *ω*) describes the bandpass function of the rotationally-scanned ISAM system. The function *κ͌*(*ξ*, *η_θ_*, *ω*) is a Fourier transform of a resampled version of the Fourier transform of the object being sought. That is,

(36) $$\overset{\approx }{\kappa }(\xi ,\eta_{\theta },\omega )=\int_{-\pi }^{\pi }d\theta \;\exp(i\theta \eta_{\theta })\;{\overset{\approx }{\eta }}^{\prime \prime }\left\{-\left[\hat{x}\;\cos\theta \sqrt{4k^{2}(\omega )-\xi^{2}}+\hat{y}\xi +\hat{z}\;\sin\theta \sqrt{4k^{2}(\omega )-\xi^{2}}\right]\right\},$$

where *x̂*, *ŷ*, *ẑ* are unit vectors and

(37) $${\overset{\approx }{\eta }}^{\prime \prime }(q)=\int d^{3}r\;\exp(ir\cdot q)\frac{\eta (r)}{P(r)},$$

is a weighted Fourier transform of the object *η*(**r**), with *P*(**r**) a function of the radial distance to focus [16]. It is thus seen that the solution of inverse problem may again be reduced to a filtering and resampling of the data between appropriate Fourier transforms and inverse Fourier transforms.

### 7.4. Partially-Coherent ISAM

In a recent analysis [97], it has been shown that a spatially-extended, statistically partially-coherent source can be incorporated in full-field ISAM to produce differing illumination and detection patterns *g*(**r**, *ω*) and *f* (**r**, *ω*). Varying the source coherence length allows considerable control of *g*(**r**, *ω*) and also results in a changing resampling scheme in the inverse problem. The multiple scattering artifacts that can be problematic in full-field ISAM can be effectively mitigated using partially-coherent ISAM.

## 8. Conclusions

ISAM is a computed imaging technique that quantitatively estimates a three-dimensional scattering object in broadband coherent microscopy. The solution of the inverse problem allows the reconstruction of areas typically regarded as out of focus. The result obviates the perceived trade-off between resolution and depth of focus in OCT.

ISAM, like OCT, is a tomographic method, i.e., the images produced are truly three-dimensional. While ISAM addresses an inherent weakness in OCT, namely the need to scan the focus axially to obtain images outside of the original focal plane, ISAM is not merely a method to refocus the field computationally. Refocusing may be achieved from a single interferometric image at a fixed frequency, but the resulting image is still inherently two-dimensional, failing to unambiguously distinguish contributions to the image from various depths. As in other ranging technologies, the broadband nature of ISAM allows a true three-dimensional reconstruction.

ISAM and SAR are closely related technologies, to the point where they can be cast in the same mathematical framework. Both techniques employ a Fourier domain resampling, based on the Stolt mapping, in the inverse processing. While the mathematics of the two systems are closely related, each uses a significantly different region of the electromagnetic spectrum and images objects of com-mensurately different scales. In SAR, translation of the aperture and computational imaging allow the synthesis of a virtual aperture of dimension dependent on the along track path length, rather than the physical aperture size. This larger synthetic aperture produces an image of higher resolution than would otherwise be achievable. In OCT the limitations on the size of the physical aperture (i.e., the objective lens) are not the limiting factor, rather the image acquisition time becomes prohibitively long if the focal plane must be scanned through an object of extended depth. The computational imaging in ISAM gives diffraction-limited resolution in all planes, not just at the physical focus, and hence eliminates the need for focal-plane scanning.

ISAM and SAR are examples in the broad class of modalities known as computed imaging. Like almost all computed imaging modalities in common practice today, they are based on the solution of linear inverse problems. Linear inversion problems offer advantages such as the option to pre-compute and store the elements of an inversion kernel for rapid computation of images from data. Moreover, error and stability may be well understood and there exist a wealth of well-studied methods for regularization (stabilization) of the inversion algorithms. ISAM and SAR are also members of the more restrictive class of problems that may be cast as data resampling. To arrive at the resampling view of these problems, the data must be Fourier transformed and the resampled data Fourier transformed again. Thus the methods take advantage (are even reliant on) one of the greatest advances in applied mathematics in the last half-century, the fast Fourier transform [98]. They may be made to run very fast and are amenable to parallelization.
